# Voxel-based Three-dimensional Segmentation of the Capsulo-synovium from Contrast-enhanced MRI Can Represent Clinical Impairments in Adhesive Capsulitis

**DOI:** 10.1038/s41598-020-63406-9

**Published:** 2020-04-16

**Authors:** Jung-Sang Lee, Jong Geol Do, Kyung Jae Yoon, Seoung Wan Chae, Hee-Jin Park, Chul-Hyun Park, Yong-Taek Lee

**Affiliations:** 1 Department of Physical and Rehabilitation Medicine, Kangbuk Samsung Hospital, Sungkyunkwan University School of Medicine, Seoul, Republic of Korea; 2 Department of Pathology, Kangbuk Samsung Hospital, Sungkyunkwan University School of Medicine, Seoul, Republic of Korea; 3 Department of Radiology, Kangbuk Samsung Hospital, Sungkyunkwan University School of Medicine, Seoul, Republic of Korea; 4 Department of Physical and Rehabilitation Medicine, Samsung Medical Center, Sungkyunkwan University School of Medicine, Seoul, Republic of Korea

**Keywords:** Musculoskeletal abnormalities, Tendons

## Abstract

The purposes were to calculate total voxel volume of the entire capsulo-synovial enhanced portion on contrast-enhanced (CE) MRI in adhesive capsulitis, and to investigate its association with glenohumeral joint volume and passive range of motions (ROMs), which are a well-known diagnostic reference standard and clinical hallmark of this condition. Medical records of 169 consecutive patients who underwent ultrasound-guided intraarticular injection with adhesive capsulitis and CE-MRI to exclude other mimicking shoulder diseases were retrospectively reviewed. To calculate total voxel volume of entire capsulo-synovial enhanced portion on CE-MRI, voxel-based 3-dimensional (3D) segmentation was obtained semi-automatically using Fiji, an open-source image processing software. Pearson’s correlation coefficients were analyzed. Sixty patients who met eligibility criteria were included. Total voxel volume showed a significant inverse correlation with the glenohumeral joint volume (*r* = −0.528, *P* < 0.001), forward elevation, external rotation, and abduction (*r* = −0.407, *P* = 0.001; r = −0.342, P = 0.007; r = −0.275, P = 0.034, respectively). Intra-observer and inter-observer reliabilities, measured by intraclass correlation coefficients (ICC), were excellent (ICC = 0.87 and 0.77, respectively). This study’s results indicate that voxel-based 3D segmentation of entire capsulo-synovial enhanced portion from CE-MRI can represent the severity of clinical impairments, such as obliterated joint volume and limited passive ROMs in adhesive capsulitis.

## Introduction

**Figure 1:**
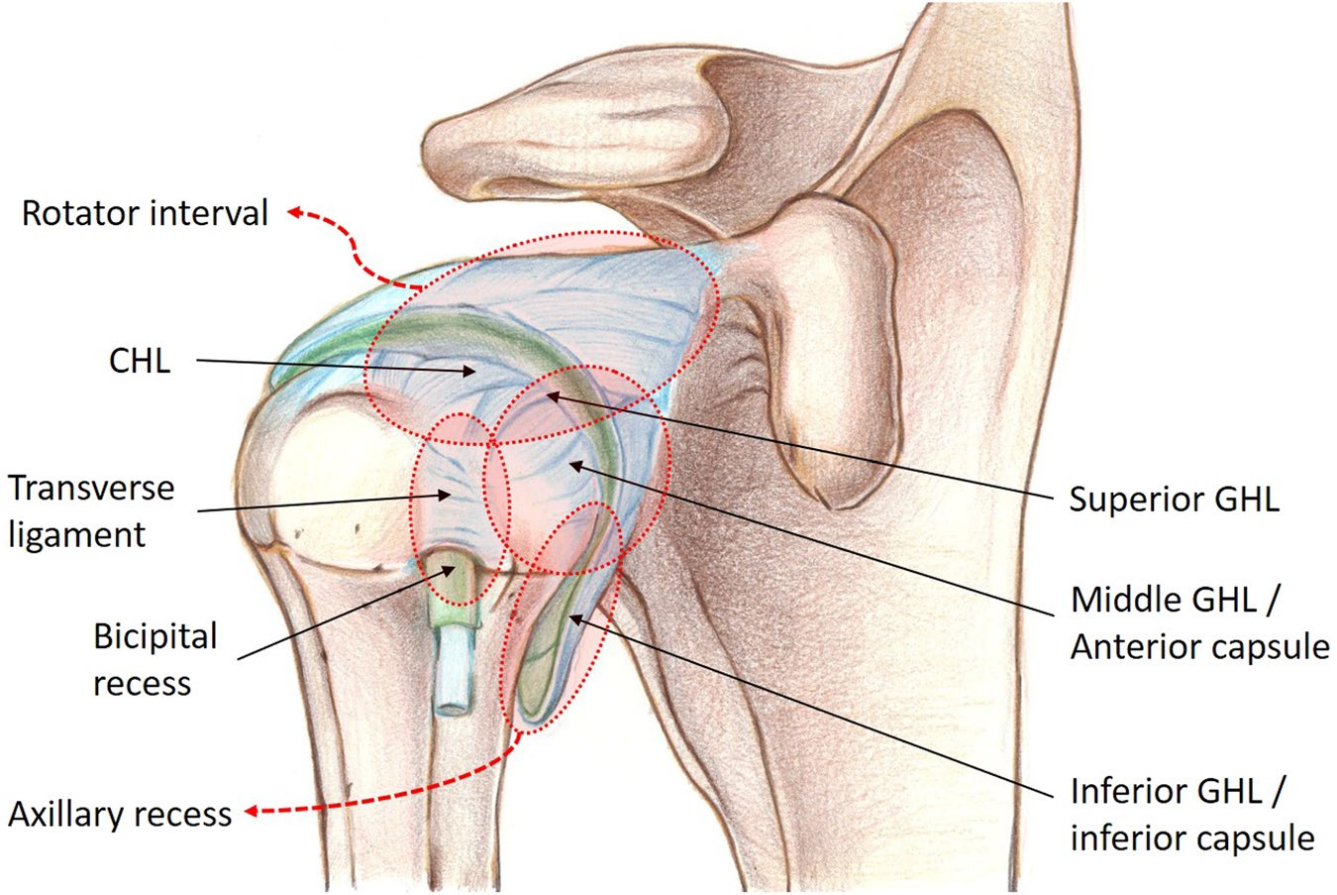
Anatomical structures that pathologic changes can involve with varying degree of severity according to the disease stage in adhesive capsulitis. CHL = coracohumeral ligament; GHL = glenohumeral ligament.

Adhesive capsulitis is characterized by a painful and gradual loss of both active and passive range of motions (ROMs) of the glenohumeral joint, which is thought to result from a combination of inflammation and fibrosis of the capsule-synovium^1,2^. The incidence in the general population is approximately 3% to 5% but as high as 20% in patients with diabetes^2^. This condition is primarily diagnosed clinically, but its correct diagnosis is difficult to make because other shoulder conditions have similar clinical symptoms. Thus, imaging studies such as magnetic resonance imaging (MRI) can be complementary and helpful to exclude other causes of a painful stiff shoulder. In the literature, several MRI findings such as capsulo-synovial thickness in the axillary recess^3–6^, thickness of the coracohumeral ligament (CHL)^7^, width of the rotator interval^5,8^, and thickness of the capsulo-synovial enhanced portion in the axillary recess^9–11^ or rotator interval^9,10^ have been reported as reliable signs for diagnosing this condition. However, debates remain because other studies have shown controversial results^3,4,7,11,12^. These controversial results may be because previous studies used a one-dimensional measurement (i.e., thickness, width, etc.) at the pre-designated localized region including axillary recess, CHL, or rotator interval etc. although pathologic changes in adhesive capsulitis can involve any anatomical structures in the global shoulder joint with varying degrees of severity according to the disease stage (Fig. 1)^1,9,13,14^. Thus, we hypothesized that a three-dimensional (3D) quantification of the entire capsulo-synovial enhanced portion would provide reliable and exact information about the pathologic status of the joint in adhesive capsulitis. The purposes of this study were to calculate the total voxel volume of the entire capsulo-synovial enhanced portion on contrast-enhanced (CE)-MRI in adhesive capsulitis, and to investigate its association with the glenohumeral joint volume and passive ROMs, which are a well-known diagnostic reference standard and clinical hallmark of this condition, respectively.

## Materials and Methods

### Participants

**Figure 2:**
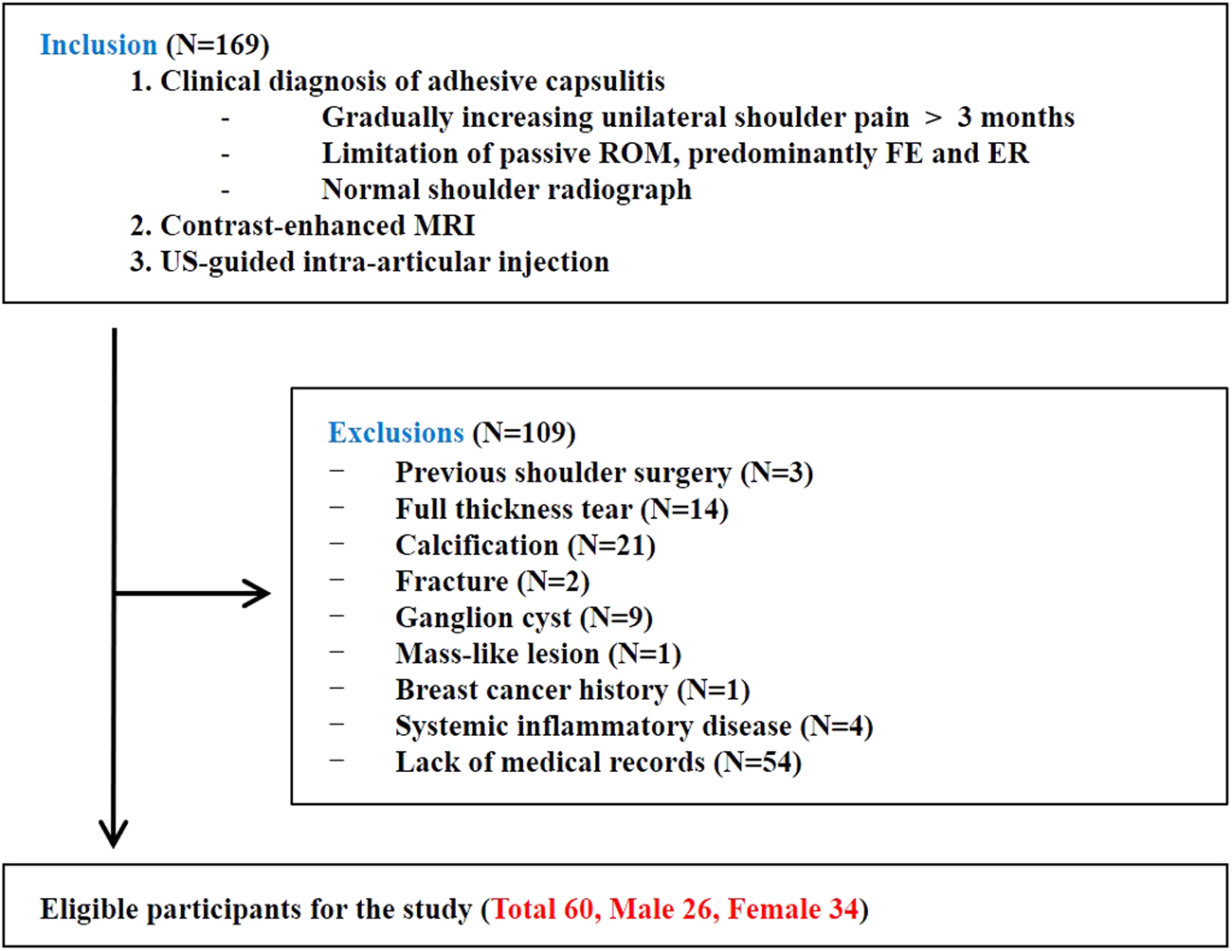
Flow chart for selection of the eligible study subjects. ROM = range of motion; FE = forward elevation; ER = external rotation.

We reviewed the medical records of 169 consecutive patients who presented to the outpatient shoulder clinic of our department with a clinical diagnosis of adhesive capsulitis and who underwent ultrasound-guided intra-articular injection and CE-MRI to exclude other mimicking shoulder diseases between January 2007 and July 2017. CE-MRIs with appropriate Digital Imaging and Communications in Medicine (DICOM) image data source for image processing were included. Diagnosis of adhesive capsulitis was based on the criteria modified from the previous studies^9,10^ by an experienced physiatrist (Y.T.L., 15 years of experience in musculoskeletal field) as follows: (1) gradually increasing unilateral shoulder pain for at least 3 months, (2) limitation of the shoulder passive ROM predominantly at forward elevation and external rotation, and (3) normal glenohumeral joint and acromio-humeral joint radiographs. Patients who had secondary causes such as calcific tendinitis (n = 21) or full-thickness tear of the rotator cuff (n = 14) were excluded. Additionally, previous shoulder surgery (n = 3), shoulder fracture (n = 2), ganglion cyst (n = 9), mass-like lesion (n = 1), breast cancer history (n = 1), systemic inflammatory disease (n = 4), or lack of medical records (n = 54) were excluded. Finally, sixty patients (26 men and 34 women) who met our eligibility criteria were included in this study (Fig. 2). Table 1 summarizes the basic characteristics. All data were gathered retrospectively by review of medical records. Institutional Ethics Review Board of Kangbuk Samsung Hospital approved this study and waived the requirement for informed consent because of the retrospective study design (KBSMC 2016-08-060). All methods were performed in accordance with the relevant guidelines and regulations.

**Table 1:** Basic characteristics of the subjects.

Characteristics	All Patients (N = 60)
Female, n (%)	34 (56.7%)
Age, years	56.4 ± 9.0
Disease duration, months	6.6 ± 9.2
Total voxel volume, mm^3^	1154.1 ± 990.9
GHJ volume, mL	6.6 ± 3.3
Passive ROM, degree	
Forward elevation	137.1 ± 28.6
External rotation	22.4 ± 25.6
Abduction	89.9 ± 40.9

GHJ = glenohumeral joint; ROM = range of motion.

Additional Information.

### CE-MRI protocol

MR examination of the shoulder were performed using a 1.5 T MR scanner (Signa HDxt, GE Healthcare, Milwaukee, WI, USA) with an 8-channel shoulder-dedicated coil with patients in a neutral upper arm position. For CE-MRI, T1-weighted images with fat-suppression after injection of 0.1 mmol/kg of gadobutrol or 0.1 mmol/kg of gadodiamide were obtained. MRI parameters were as follows: field of view, 14–16 cm; matrix size, 304 × 240–340 × 257; repetition time/echo time, 541-758/9-25; slice thickness, 3.0 or 4.0 mm; interslice gap, 0.4 or 1.0 mm; in-plane resolution, 0.27 × 0.27–0.39 × 0.39 mm; and voxel depth, 4.0, 4.4 or 5.0 mm.

### Calculation of the total voxel volume

**Figure 3:**
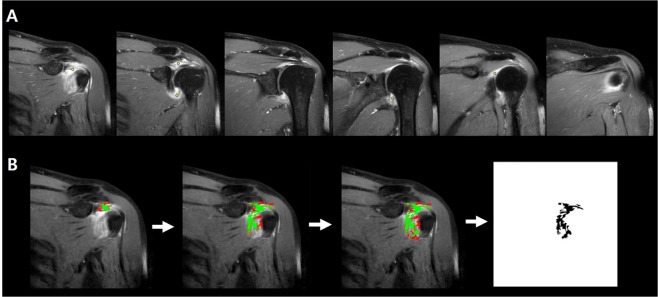
Semiautomatic segmentation of the regions of interests (ROIs) using the Fast Marching method of Level Set plugin (https://imagej.net/Level_Sets) in Fiji, an open-source image processing package based on Image J. (**A)** Before image segmentation, all oblique coronal fat-suppressed enhanced T1-weighted images were imported to the Fiji software and the image dynamic was reduced from 16-bit to an 8-bit type to match the requirements of the used software. In the Fiji environment, the operator manually selected several seed points on the capsulo-synovial enhanced portion in the serial MR image stack. Capsulo-synovial enhanced portion was defined as the area where signal intensity was enhanced and regarded as pathologic condition. (**B**) Then, we went to the Level Sets plugin dialog and selected the Fast Marching option. Clinking on OK button, Fiji automatically updated progress window (the first, second and third figures) and, after completion, showed a result window (the fourth figure). The segmented area was shown in green in the progress window and black in the result window. To detect segmentation boundaries, the Fast Marching method algorithm expands from a seed point to the segmentation boundary until it encounters a pre-set grey value difference in the pixel intensity. While growing the region of interest (ROI), it constantly calculates the difference of the current selection to the newly added pixels and either stops if it exceeds a pre-selected grey value difference. The grey level value of each pixel is in the range of 0-255. In this study, the threshold of grey value difference between boundary pixel and the seed point was pre-set with 50, which determines the stopping point for the expansion.

**Figure 4:**
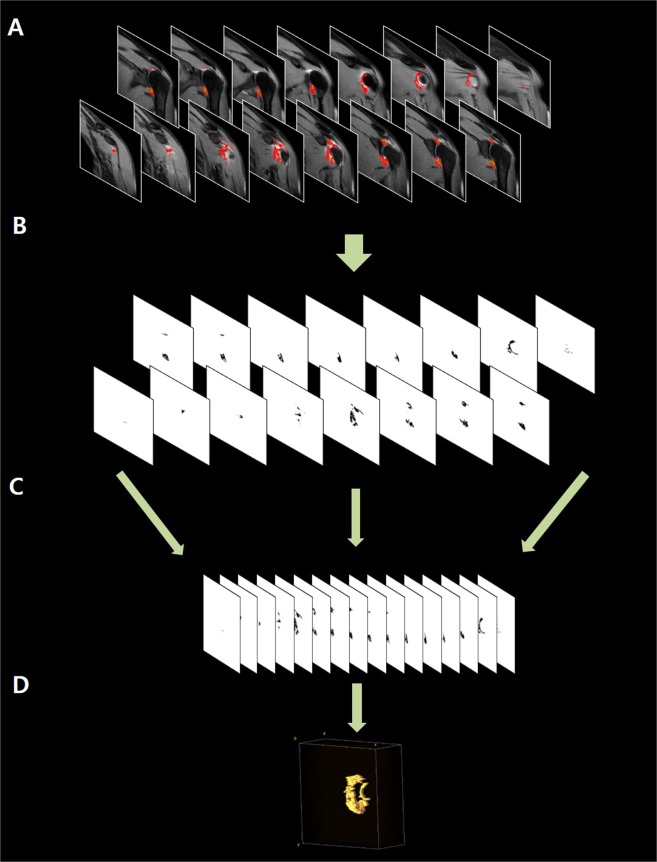
Calculation of total voxel volume and 3D reconstruction of the entire capsulo-synovial enhanced portion on CE-MRI. (**A)** Regions of interests (ROIs) are automatically created on the capsulo-synovial enhanced portion in all consecutive MR images in the progress windows by the Fast Marching method of Level Set plugin. (**B**) Segmented area of capsulo-synovial enhanced portion in all consecutive MR images are shown in the result windows by the Fast Marching method of Level Set plugin. (**C**) Subsequently, using the Voxel Counter plugin, total voxel volume of segmented area of entire capsulo-synovial enhanced portion was automatically calculated in mm^3^. (**D**) Finally, 3D shape of the segmentation of entire capsulo-synovial enhanced portion was reconstructed and illustrated by the Volume Viewer plugin.

**Figure 5:**
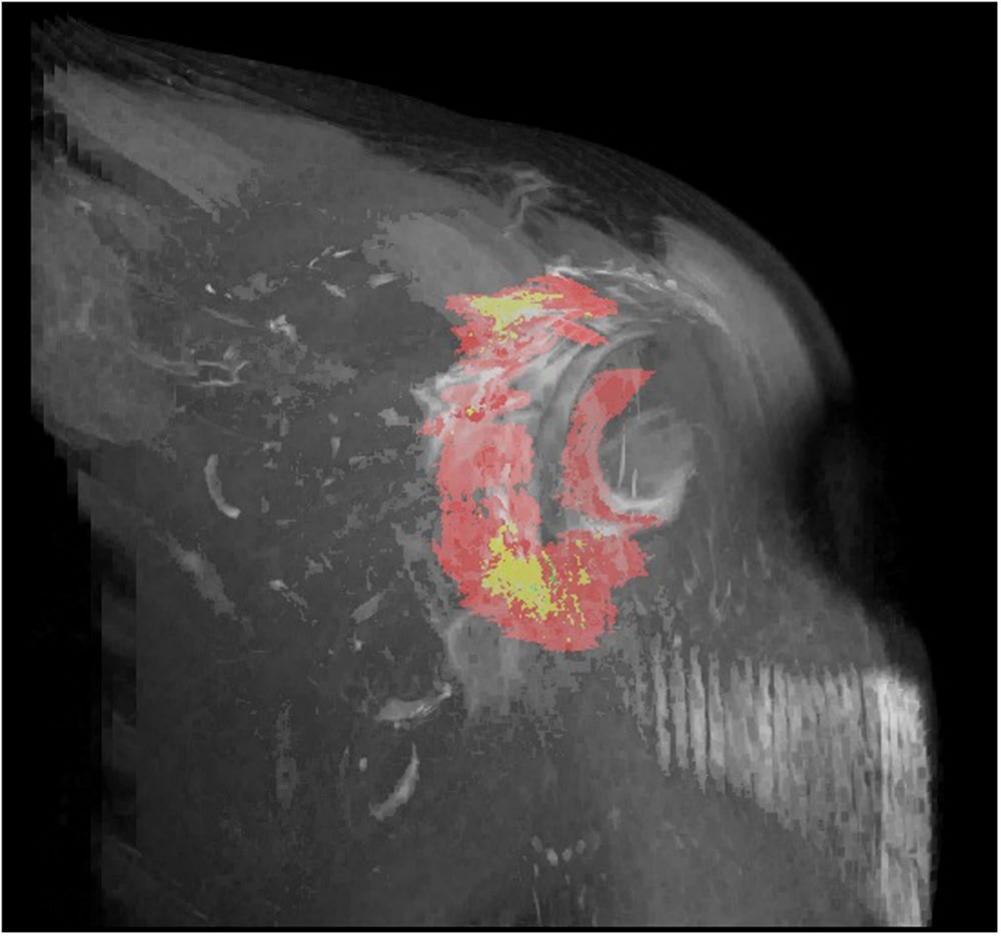
The segmentation of the entire capsulo-synovial enhanced portion of the shoulder joint in adhesive capsulitis was 3-dimensionally reconstructed and illustrated using the 3D Project function in Fiji (supplement material Video 1).

Calculation of the total voxel volume was conducted by two of the authors (C.H.P. and J.S.L., 3 years of experience in musculoskeletal field, respectively). To calculate total voxel volume of the entire capsulo-synovial enhanced portion on CE-MRI, the voxel-based 3D segmentation was obtained semi-automatically using the Fast Marching method of Level Set plugin (https://imagej.net/Level_Sets) in Fiji, an open-source image processing package based on Image J^15^. Before image segmentation, all oblique coronal fat-suppressed enhanced T1-weighted images were imported to the Fiji software and the image dynamic was reduced from 16-bit to an 8-bit type to match the requirements of the used software. In the Fiji environment, the operator manually selected several seed points on the capsulo-synovial enhanced portion in the serial MR image stack (Fig. 3A). Capsulo-synovial enhanced portion was defined as the area where signal intensity was enhanced and regarded as pathologic condition. Then, we went to the Level Sets plugin dialog and selected the Fast Marching option. Clinking on OK button, Fiji automatically updated progress window (the first, second and third figures in Figs. 3B, 4A) and, after completion, showed a result window (the fourth figure in Figs. 3B, 4B). The segmented area was shown in green in the progress window and black in the result window. To detect segmentation boundaries, the Fast Marching method algorithm expands from a seed point to the segmentation boundary until it encounters a pre-set grey value difference in the pixel intensity. While growing the region of interest (ROI), it constantly calculates the difference of the current selection to the newly added pixels and either stops if it exceeds a pre-selected grey value difference. The grey level value of each pixel is in the range of 0–255. In this study, the threshold of grey value difference between boundary pixel and the seed point was pre-set with 50, which determines the stopping point for the expansion. Subsequently, using the Voxel Counter plugin, total voxel volume of segmented area of entire capsulo-synovial enhanced portion was automatically calculated in mm^3^ (Fig. 4C). Finally, 3D shape of the segmentation of entire capsulo-synovial enhanced portion was reconstructed and illustrated by the Volume Viewer plugin (Fig. 4D) and the 3D Project function in Fiji (Fig. 5; supplement material Video 1).

### Reliability of the total voxel volume

For intra-observer reliability, one of the authors (J.S.L.) calculated total voxel volume twice, at 3-week intervals. Second investigator (C.H.P.) calculated the total voxel volume to compare with that calculated by the first investigator (J.S.L.) for inter-observer reliability.

### Measurement of the glenohumeral joint volume

All the sono-guided intra-articular injection were conducted mean 3.2 weeks (0–26 weeks) after MRI scan by an experienced physiatrist (Y.T.L.). The glenohumeral joint volume have been measured and recorded during sono-guided intra-articular injection by the same person who conducts injection in our routine clinical practice. Under high-resolution ultrasound guidance (Voluson 730 Pro, GE Healthcare, Milwaukee, WI, USA; RS80A, Samsung Medicine, Gyeonggi-do, Republic of Korea) with a posterior approach, 20-mL of a solution consisting of corticosteroids, local anesthetics, and normal saline was injected with gentle pressure into the shoulder joint. We used the same size needle (23 gauge, 6 cm) and syringe (20 mL) in all patients. The injection was ceased immediately when the rebound resistance of the plunger was felt or when the patient complained of pain preventing further injection during the procedure. The amount of volume injected into the shoulder joint was regarded as the glenohumeral joint volume. Proper intra-articular injection was confirmed by observing distension of the posterior joint capsule by ultrasonography.

### Measurement of Passive ROMs

Passive ROMs was also measured and recorded before sono-guided intra-articular injection by the same physician who conducted injection (Y.T.L.). Using a goniometer, shoulder passive ROMs comprising forward elevation, external rotation at the side and abduction were measured with the patient sitting on a stool before the sono-guided intra-articular injection^16^. Forward elevation was measured by the maximum arm trunk angle when the examiner elevated the patient’s upper arm in the sagittal plane of the trunk. External rotation was measured with the patient’s arm in close adduction at the trunk and with the elbow at 90° of flexion. Abduction was measured by the maximum arm trunk angle when the examiner elevated the patient’s upper arm in the coronal plane of the trunk. When evaluating passive ROM, we pressed down on the patient’s scapula firmly and asked the patient to relax as much as possible to reduce the influence of compensatory movement of the spine and the scapula.

### Data analysis

We used Pearson’s correlation coefficient (r) to assess the association between the two variables, i.e. between the total voxel volume and glenohumeral joint volume; between the total voxel volume and forward elevation; between the total voxel volume and external rotation; between the total voxel volume and abduction. The intraclass correlation coefficient (ICC) was used to evaluate intra-observer (J.S.L.) and inter-observer (C.H.P. and J.S.L.) reliabilities for the calculation of the total voxel volume of the entire capsulo-synovial enhanced portion. The ICC was rated as follows: >0.75, excellent agreement; 0.40–0.75, good agreement; 0.20–0.40, fair agreement; and 0.00–0.20, poor agreement. All statistical analyses were performed using SPSS for Windows software, version 18.0 (SPSS), and *P*-values < 0.05 were considered statistically significant.

## Results

The mean total voxel volume of the entire capsulo-synovial enhanced portion was 1154.1 ± 990.9 mm^3^. The mean glenohumeral joint volume was 6.6 ± 3.3 mL. Regarding mean passive ROMs of the shoulder, forward flexion, external rotation, and abduction were 137.1 ± 28.6°, 22.4 ± 25.6°, and 89.9 ± 40.9°, respectively (Table 1).

**Figure 6:**
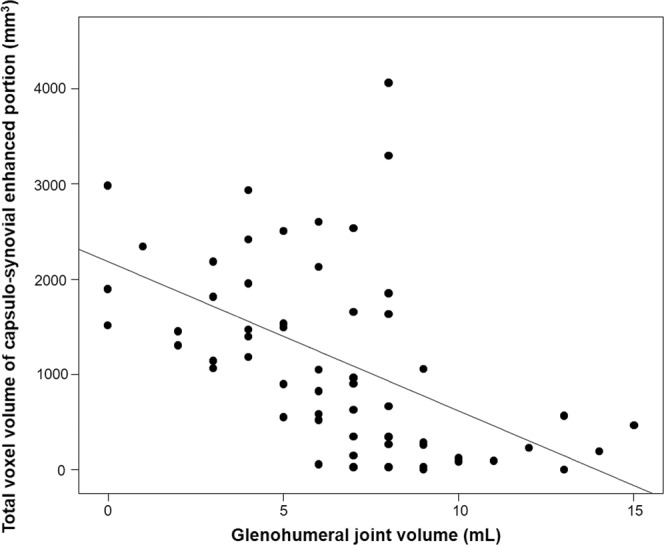
The mean total voxel volume of the entire capsulo-synovial enhanced portion showed a significant inverse correlation with the glenohumeral joint volume of shoulder by Pearson’s correlation coefficient (*r* = −0.528, *P* < 0.001).

The total voxel volume of the entire capsulo-synovial enhanced portion was inversely correlated with the glenohumeral joint volume (*r* = −0.528, *P* < 0.001) (Fig. 6). Regarding passive ROMs, the total voxel volume of the entire capsulo-synovial enhanced portion was also inversely correlated with forward elevation; external rotation; and abduction (*r* = −0.407, *P* = 0.001; r = −0.342, P = 0.007; r = −0.275, P = 0.034, respectively). Both intra-observer and inter-observer reliabilities, measured by the ICC, were excellent (ICC = 0.87 and 0.77, respectively).

## Discussion

Herein, the total voxel volume of the entire capsulo-synovial enhanced portion on CE-MRI had a good inverse correlation with the glenohumeral joint volume, which is a well-known diagnostic reference standard of adhesive capsulitis. Additionally, all passive ROM values, which are well-known clinical hallmarks of this condition, were also inversely correlated with the total voxel volume of the entire capsulo-synovial enhanced portion on CE-MRI. Both intra-observer and inter-observer reliabilities, analyzed by the ICC, were excellent. These findings indicate that the voxel-based 3D assessment of the capsulo-synovial enhancement on CE-MRI can represent severity of clinical impairments in adhesive capsulitis, such as obliterated joint volume and limitation of passive ROMs. Previous studies using non-enhanced MRI or direct MR arthrography have reported conflicting results on reliable MRI findings for the diagnosis of adhesive capsulitis. Thickness of the capsule in the axillary recess^3–5^, thickness of the CHL^7^ and width of the rotator interval^5,8^ have been described as characteristic findings of this condition. However, other similar studies have reported controversial results that thickness of the capsule in the axillary recess^7,12^, thickness of the CHL^3^, or rotator interval width^3,4^ is not useful for diagnosing adhesive capsulitis. In terms of CE-MRI, some studies reported thickening of the capsulo-synovial enhancement at the axillary recess or rotator interval^6,9,10^ are signs suggestive of adhesive capsulitis, but other study demonstrated the thickening of the capsulo-synovial enhancement at the rotator interval was not useful^11^. These controversial results may be attributed to the fact that previous studies used the one dimensional measurement (thickness or width) at a pre-designated localized anatomical region, such as axillary recess, CHL, or rotator interval, although pathologic changes in adhesive capsulitis can involve any anatomical structure in the shoulder joint with varying degrees of severity (Fig. 1)^1,9,13,14^. The pathologic change of adhesive capsulitis is thought to be cytokine-mediated synovial inflammation followed by capsular fibrosis^14^. In the initial stage, hypervascular synovial hyperplasia caused by inflammation is observed, and subsequent fibrosis in the capsule and sub-synovium results from collagen accumulation. In the later stages, as the inflammation subsides, capsular fibrosis increases, leading to thickening or contracture of the capsule-synovium and the surrounding ligamentous structures^2,13,14^. These pathologic changes can involve not only a specific localized area but also any anatomical structure in the shoulder joint with varying degrees of severity according to the disease stage^1,9,13,14^. Thus, 3D assessment of the entire pathologic lesion in the shoulder joint could provide more reliable and exact information than one dimensional measurement at a designated localized area in  which previous studies conducted.

The present study is the first to obtain the voxel-based 3D segmentation of the capsulo-synovial enhanced portion, as previous MRI studies addressed a one-dimensional measurement (i.e., thickness, width, etc.) at a pre-designated localized region^3–11^. Recently, Ogul *et al*. studied 3D volumetric assessment of joint capacity on direct MR arthrography. They suggested that obliterations in the biceps tendon sheath and rotator interval as well as decreased joint capacity on 3D volumetric MR arthrographic examination are another useful criteria in diagnosing primary adhesive capsulitis^17^. However, thickening and contrast enhancement of capsule/synovium by pathologic changes can be demonstrated more obviously by CE-MRI, which is mainly due to increased vascularity in and around these structures^9,10,18,19^. The quantity of enhancement varies by the varying degree of vascularity and amount of fibrotic tissue according to the disease stage^16^. Although direct MR arthrography with intra-articular contrast injection can demonstrate specific imaging findings for adhesive capsulitis, such as thickened and contracted axillary recess, rotator interval and biceps tendon sheath, it mainly demonstrates a contour of anatomical structure and joint capacity by the injected contrast material into intraarticular space but cannot show the degree of vascularity of the joint capsule and synovium. Thus, our study is unique because we used CE-MRI with intravenous contrast injection for the 3D assessment of pathologic changes of capsule/synovium itself throughout the shoulder joint.

Obtaining the 3D segmentation in a complex anatomic structure such as the shoulder joint is a challenging task because irregular margins of surrounding structures make it difficult to identify the structure clearly. Recently, a semi-automatic 3D measurement using image segmentation software has been used in structures with irregular distribution, such as visceral fat and the liver, and it showed advantages in tracing and quantifying the boundary^20,21^. We used the Fast Marching method of Level Sets, the semi-automatic segmentation plugin in Fiji, which is less time-consuming and less observer-dependent because it automatically segments the ROIs with similar intensity of brightness (i.e. within grey value difference 50 in our study) between the boundary pixel and the seed point that the operator manually selects in consecutive MR images (Fig. 3). Actually, this method revealed excellent intra-observer and inter-observer reliabilities in our study.

In our study, the mean glenohumeral joint volume was 6.6 ± 3.3 ml, which was determined by the amount of volume injected into the shoulder joint under ultrasonography (US) guide until the rebound resistance of the plunger was felt or the patient complained of pain preventing further injection. Additionally, the patients with full thickness tear that can increase the injected volume were excluded. This finding is in accord with previous reports that the capacity of the glenohumeral joint measured by conventional arthrography is obliterated under 7–10 ml in adhesive capsulitis^22^. Adhesive capsulitis has normally 4 clinical stages: preadhesive, freezing, frozen, and thawing^23^. The ‘freezing’ stage presents with increasing pain and stiffness lasting between 3 and 9 months. The ‘frozen’ stage is characterized by painful stiffening and significant loss of ROM for period lasting between 9 and 14 months. The patients in our study were likely to have the freezing stage or frozen stage because we included the patients with clinical diagnosis of adhesive capsulitis who had gradually increasing unilateral shoulder pain for at least 3 months, and limitation of the shoulder passive ROM predominantly at forward elevation and external rotation. Park et al. reported in their study with 75 subjects that mean joint space capacity were 7.1 ± 1.8 in the freezing stage and 6.5 ± 1.1 in the frozen stage, respectively^24^.

Ultrasonography (US) is the most commonly selected option for investigating shoulder disease because of its easy accessibility and cost-effectiveness. In terms of adhesive capsulitis, it has been reported that US findings can also provide useful information about soft tissue change including thickness of the CHL^25^, presence of fibro-inflammatory soft tissue^26^, and thickness of the inferior glenohumeral capsule^27^. However, US has an inherent limitation in diagnosing adhesive capsulitis because it has difficulty evaluating intraarticular structures and a narrower field of view than MRI, which make it difficult to exclude intraarticular problems and determine the entire capsulo-synovial pathology throughout the joint.

This study has several limitations that should be considered when interpreting our findings. First, the semi-automatic segmentation method used for calculating the total voxel volume of the entire capsulo-synovial enhanced portion is still difficult to standardize and time-consuming. Further, although the inter-observer agreement of the measurements was excellent, it is still operator-dependent because the pre-determined initial seed points are manually selected by the examiner. Thus, it is currently unlikely to be widely adopted in clinical practice. Further study to improve these limitations would be needed to apply this 3D assessment concept to routine clinical practice. Second, the Level Sets plugin in Fiji software has an inherent limitation, as it changes the information of the raw serial MR images from 16 bits to 8 bits before analysis. Further investigation with software that utilizes the original image would be beneficial in maintaining image resolution and reflecting more accurate analytical data. Third, MRI protocol used in this study was heterogenous because of our retrospective study design. Although those parameters, such as slice thickness and voxel depth etc., were calibrated when we calculated the total voxel volume using the Voxel Counter plugin, it could have some impact on our results. Fourth, it is well known that intra-articular injection of fluid often results in spilling of fluid in the adjacent soft tissues. Therefore, the direct intra-articular injection of fluid probably has a limited accuracy for determining glenohumeral joint volume. Finally, retrospective data collection through chart reviews can be incomplete because of a lack of medical records.

In conclusion, this study’s results indicate that the voxel-based 3D segmentation of the entire capsule-synovial enhanced portion from CE-MRI can represent the severity of clinical impairments, such as obliterated joint volume and limitation of passive ROMs, in adhesive capsulitis.

## Supplementary information

Supplementary information

## References

[1] Neviaser AS, Hannafin JA (2010). Adhesive capsulitis: a review of current treatment. The American journal of sports medicine.

[2] Le HV, Lee SJ, Nazarian A, Rodriguez EK (2017). Adhesive capsulitis of the shoulder: review of pathophysiology and current clinical treatments. Shoulder & elbow.

[3] Emig E, Schweitzer ME, Karasick D, Lubowitz J (1995). Adhesive capsulitis of the shoulder: MR diagnosis. AJR. American journal of roentgenology.

[4] Lee MH (2003). Adhesive capsulitis of the shoulder: diagnosis using magnetic resonance arthrography, with arthroscopic findings as the standard. Journal of computer assisted tomography.

[5] Jung JY (2006). Adhesive capsulitis of the shoulder: evaluation with MR arthrography. Eur Radiol.

[6] Song KD, Kwon JW, Yoon YC, Choi S-H (2011). Indirect MR arthrographic findings of adhesive capsulitis. American Journal of Roentgenology.

[7] Mengiardi B, Pfirrmann CW, Gerber C, Hodler J, Zanetti M (2004). Frozen shoulder: MR arthrographic findings. Radiology.

[8] Kim KC, Rhee KJ, Shin HD (2009). Adhesive capsulitis of the shoulder: dimensions of the rotator interval measured with magnetic resonance arthrography. Journal of shoulder and elbow surgery.

[9] Gokalp G, Algin O, Yildirim N, Yazici Z (2011). Adhesive capsulitis: contrast-enhanced shoulder MRI findings. Journal of medical imaging and radiation oncology.

[10] Lefevre-Colau MM (2005). Magnetic resonance imaging of shoulders with idiopathic adhesive capsulitis: reliability of measures. Eur Radiol.

[11] Lee, Y.-T. *et al*. Correlation of Joint Volume and Passive Range of Motion With Capsulo-Synovial Thickness Measured by Contrast-Enhanced Magnetic Resonance Imaging in Adhesive Capsulitis. *PM&R* (2017). 10.1016/j.pmrj.2017.06.023 28729060

[12] Manton GL, Schweitzer ME, Weishaupt D, Karasick D (2001). Utility of MR arthrography in the diagnosis of adhesive capsulitis. Skeletal radiology.

[13] Uhthoff HK, Boileau P (2007). Primary frozen shoulder: global capsular stiffness versus localized contracture. Clinical orthopaedics and related research.

[14] Hsu JE, Anakwenze OA, Warrender WJ, Abboud JA (2011). Current review of adhesive capsulitis. Journal of shoulder and elbow surgery.

[15] Schindelin J (2012). Fiji: an open-source platform for biological-image analysis. Nature methods.

[16] Ahn KS, Kang CH, Oh YW, Jeong WK (2012). Correlation between magnetic resonance imaging and clinical impairment in patients with adhesive capsulitis. Skeletal radiology.

[17] Ogul H (2014). Extra-articular contrast material leaks into locations unrelated to the injection path in shoulder MR arthrography. Eur Radiol.

[18] Carrillon Y, Noel E, Fantino O, Perrin-Fayolle O, Tran-Minh VA (1999). Magnetic resonance imaging findings in idiopathic adhesive capsulitis of the shoulder. Revue du rhumatisme (English ed.).

[19] Tamai K, Yamato M (1997). Abnormal synovium in the frozen shoulder: a preliminary report with dynamic magnetic resonance imaging. Journal of shoulder and elbow surgery.

[20] Addeman BT (2015). Validation of volumetric and single‐slice MRI adipose analysis using a novel fully automated segmentation method. Journal of Magnetic Resonance Imaging.

[21] Yang X (2014). A hybrid semi-automatic method for liver segmentation based on level-set methods using multiple seed points. Computer methods and programs in biomedicine.

[22] Loyd JA, Loyd HM (1983). Adhesive capsulitis of the shoulder: arthrographic diagnosis and treatment. Southern medical journal.

[23] Hannafin, J. A. & Chiaia, T. A. Adhesive capsulitis. A treatment approach. *Clinical orthopaedics and related research*, 95-109 (2000). 10738419

[24] Park G-Y, Park JH, Kwon DR, Kwon DG, Park J (2017). Do the Findings of Magnetic Resonance Imaging, Arthrography, and Ultrasonography Reflect Clinical Impairment in Patients With Idiopathic Adhesive Capsulitis of the Shoulder?. Archives of Physical Medicine and Rehabilitation.

[25] Homsi C, Bordalo-Rodrigues M, da Silva JJ, Stump XM (2006). Ultrasound in adhesive capsulitis of the shoulder: is assessment of the coracohumeral ligament a valuable diagnostic tool?. Skeletal Radiol.

[26] Lee JC, Sykes C, Saifuddin A, Connell D (2005). Adhesive capsulitis: sonographic changes in the rotator cuff interval with arthroscopic correlation. Skeletal radiology.

[27] Michelin P, Delarue Y, Duparc F, Dacher JN (2013). Thickening of the inferior glenohumeral capsule: an ultrasound sign for shoulder capsular contracture. European radiology.

